# *Francisella novicida*-Containing Vacuole within *Dictyostelium discoideum*: Isolation and Proteomic Characterization

**DOI:** 10.3390/microorganisms12101949

**Published:** 2024-09-26

**Authors:** Valentina Marecic, Olga Shevchuk, Marek Link, Ina Viduka, Mateja Ozanic, Rok Kostanjsek, Mirna Mihelcic, Masa Antonic, Lothar Jänsch, Jiri Stulik, Marina Santic

**Affiliations:** 1 Department of Microbiology and Parasitology, Faculty of Medicine, University of Rijeka, 51000 Rijeka, Croatia; vmrvcic@gmail.com (V.M.); ina.viduka@medri.uniri.hr (I.V.); matejaozanic@gmail.com (M.O.); mirna.mihelcic@medri.uniri.hr (M.M.); masa.antonic@uniri.hr (M.A.); 2 Department of Immunodynamics, Institute of Experimental Immunology and Imaging, University Hospital Essen, 45147 Essen, Germany; olga.shevchuk@uni-due.de; 3 Department of Molecular Pathology and Biology, Faculty of Military Health Sciences, University of Defence, 500 01 Hradec Kralove, Czech Republic; marek.link@unob.cz (M.L.); jiri.stulik@unob.cz (J.S.); 4 Department of Biology, Biotechnical Faculty, University of Ljubljana, 1000 Ljubljana, Slovenia; rok.kostanjsek@bf.uni-lj.si; 5 Helmholtz Centre for Infection Research, 38124 Braunschweig, Germany; lothar.jaensch@helmholtz-hzi.de; 6 Department of Environmental Health, Teaching Institute of Public Health of Primorje-Gorski Kotar County, 51000 Rijeka, Croatia

**Keywords:** amoeba, *Dictyostelium*, *Francisella*, intracellular life, proteome, vacuole

## Abstract

*Francisella* is a highly infectious gram-negative bacterium that causes tularemia in humans and animals. It can survive and multiply in a variety of cells, including macrophages, dendritic cells, amoebae, and arthropod-derived cells. However, the intracellular life cycle of a bacterium varies depending on the cell type. Shortly after the infection of mammalian cells, the bacterium escapes the phagosome into the cytosol, where it replicates. In contrast, in the amoebae *Acanthamoeba castellanii* and *Hartmannella vermiformis*, the bacterium replicates within the membrane-bound vacuole. In recent years, the amoeba *Dictyostelium discoideum* has emerged as a powerful model to study the intracellular cycle and virulence of many pathogenic bacteria. In this study, we used *D. discoideum* as a model for the infection and isolation of *Francisella novicida*-containing vacuoles (FCVs) formed after bacteria invade the amoeba. Our results showed that *F. novicida* localized in a vacuole after invading *D. discoideum*. Here, we developed a method to isolate FCV and determined its composition by proteomic analyses. Proteomic analyses revealed 689 proteins, including 13 small GTPases of the Rab family. This is the first evidence of *F. novicida*-containing vacuoles within amoeba, and this approach will contribute to our understanding of host–pathogen interactions and the process of pathogen vacuole formation, as vacuoles containing bacteria represent direct contact between pathogens and their hosts. Furthermore, this method can be translocated on other amoeba models.

## 1. Introduction

*Francisella tularensis* is a highly infectious gram-negative bacterium that causes the zoonotic disease tularemia in humans and animals. The genus *Francisella* includes five species: *F. tularensis*, *F. philomiragia*, *F. noatunensis*, *F. hispaniensis*, and *F. novicida* [1,2]. *F. tularensis* subsp. *tularensis* and *holarctica* are highly virulent to humans [3]. Compared to *F. tularensis*, infections with *F. novicida* are not associated with healthy individuals; they are rare and, therefore, often difficult to accurately diagnose [4]. However, *F. novicida* shares homology with highly virulent subspecies and is often used as a surrogate for studying *Francisella* virulence and the pathogenesis of tularemia [1].

*Francisella* is a significant pathogen that has a high morbidity and mortality rate. The most virulent strain of *Francisella* can cause systemic forms of tularemia (pneumonic, typhoidal), with reported mortality rates of up to 60% [5,6]. The Center for Disease Control and Prevention (CDC) reports approximately 250 cases of tularemia each year. The antibiotic for treatment depends on the patient and the severity of the disease. Aminoglycosides, quinolones, tetracyclines, and chloramphenicol are frequently used in the treatment [7]. Currently, no vaccine against tularemia is available for humans.

*Francisella* has a complicated intracellular life cycle and high persistence in the environment [8]. *Francisella* is capable of infecting a variety of different hosts, including dendritic cells, neutrophils, macrophages, endothelial cells, as well as protozoa [9,10,11,12]. *Francisella* spp. are resistant to free-living amoebae, and amoebae may contribute to the survival of *Francisella* spp. in the aquatic environment [13,14]. The life cycle of *Francisella* in amoebae differs significantly from that in mammalian cells. Studies showing the intracellular growth of *Francisella* in the aquatic amoebae *Acanthamoeba castellanii* and *Hartmannella vermiformis* have suggested a role for amoebae as a natural reservoir of *Francisella* [9,13,15]. Previous investigations demonstrated that *Francisella* replicates within intracellular vacuole in *A. castellanii* and *H. vermiformis* [15,16]. In contrast, in *Dictyostelium discoideum*, *F. noatunensis* escapes the phagosome into the cytosol, where bacteria undergo extensive replication, similar to mammalian cells [17]. It is known that *F. novicida* survives and replicates within *D. discoideum*, but its intracellular localization within *Dictyostelium* has not been elucidated yet [18].

The forest soil amoeba *D. discoideum* has proven to be a useful model system to study various virulence aspects of pathogenic bacteria, including *Mycobacterium* spp, *Klebsiella pneumoniae*, *Legionella pneumophila*, *Listeria monocytogenes*, *Francisella noatunensis*, and *Francisella novicida* [17,18,19,20]. *D. discoideum* has been used to study many aspects of cell biology and made a significant contribution to our understanding of membrane and intracellular trafficking. Trafficking events are controlled by small GTPases and the machinery of the soluble N-ethylmaleimide-sensitive factor attachment protein receptor (SNARE) [21,22]. Vacuole formation and maturation are regulated by inositol phospholipids and small Rab family GTPases on the surface of the vacuole. Small GTPases regulate various endocytic compartments and recruit specific effectors as they traverse the endocytic pathway [23,24,25]. Activity of the vacuolar H+-ATPase proton pump (V-ATPase) causes the acidification of the vacuole by the translocation of protons across membranes, leading to pathogen degradation [26]. The activation of V-ATPase coincides with the rapid acidification of the vacuole compartment, which begins approximately 60 s after ingestion. As maturation progresses, lysosomal acid hydrolases, which are important for digestion, are released into the vacuolar compartment. However, to facilitate their intracellular survival and replication, intracellular pathogenic bacteria manipulate the transport and maturation of vacuoles. The *D. discoideum* genome encodes three catalytic NADPH oxidase (NOX) subunits (NOXA, NOXB, and NOXC), but there is no evidence of NOX involvement in bacterial killing by *D. discoideum* [27,28]. Microbes that persist inside the phagosome should possess mechanisms to minimize oxidative stress. In addition, *D. discoideum* has been used as a model for the isolation and proteomic characterization of vacuoles containing *Legionella* [29,30,31,32], *Salmonella* [33,34], and *Mycobacterium* [35].

Upon entry into mammalian and arthropod cells, *Francisella* reside in a membrane-bound vacuole that maintains early endosomal markers. The early vacuole compartment resists maturation and does not fuse with secondary lysosomes, as evidenced by the limited acquisition of cathepsin D and lysosome-associated membrane glycoproteins. The bacterium then escapes into the cytosol of infected cells, where it replicates [36,37,38].

For intracellular bacterial pathogens, the host cytosol provides a nutrient-rich environment. However, bacteria can colonize and proliferate in membrane-bound vacuoles in the host endomembrane system [39]. We have previously developed a method to isolate *Francisella novicida*-containing vacuoles (FCV) from primary human macrophages [40].

The aim of this study was to isolate *Francisella novicida*-containing phagosome within *D. discoideum.* We used *D. discoideum* because it is a well-established cell biology model, whose genom is completely sequenced, and which is available in dictyBase. We established a method to isolate FCV from infected *D. discoideum* cells and identified proteins associated with this compartment using LC-MS/MS proteomics. Several Rab GTPases and vacuolar ATPases were identified in FCV isolated from *D. discoideum* 60 min postinfection, and the colocalization of two selected representatives, Rab7a and VatA, with *F. novicida* was confirmed.

This is the first evidence of *F. novicida*-containing vacuoles within amoeba. The established method can be translocated to other amoeba and, thus, contribute to future research.

## 2. Materials and Methods

### 2.1. Bacterial and Cell Strains, Culture Conditions 

The wild type of *Francisella novicida* strain U112 was kindly obtained from Prof. Anders Sjöstedt (Umeå University, Umeå, Sweden). The strain was grown on buffered charcoal yeast extract (BCYE) agar at 37 °C for 24 h. BCYE agar contained 10 g of Yeast extract (Thermo Fischer Scientific, Waltham, MA, USA), 10 g of ACES Buffer (Sigma-Aldrich, St. Louis, MO, USA), 2 g of Activated Charcoal (Sigma-Aldrich, St. Louis, MO, USA), 1 g of Alpha Ketoglutarate (Sigma-Aldrich, St. Louis, MO, USA), and 20 g of Bacteriological Agar (Thermo Fischer Scientific, Waltham, MA, USA) per liter of deionized water. The pH of the medium was adjusted to 6.9. After autoclaving and cooling, 0.25 g of FeNO3 (Sigma-Aldrich, St. Louis, MO, USA) and 0.4 g of L-Cysteine (Sigma-Aldrich, St. Louis, MO, USA) were added.

The *D. discoideum* wild-type strain AX2 (gift from Michael Steinert, Technical University of Braunschweig, Braunschweig, Germany) was grown axenically in HL5 medium at 25 °C, as previously described [18]. For each experiment, *D. discoideum* cells were removed from the culture flasks, centrifuged (230× *g*, 5 min), resuspended in HL5 medium containing 1× Sorensen buffer (200×, TCS Biosciences Ltd., Buckingham, UK, 1:1), counted, washed once, and resuspended in HL5 medium containing Sorensen buffer at the desired concentration.

### 2.2. Purification of Francisella novicida-Containing Vacuole from D. discoideum

The isolation of *F. novicida*-containing vacuoles (FCV) from infected *D. discoideum* cells was performed, as previously described, and modified [31,40]. Briefly, *D. discoideum* (108 cells) were seeded in an HL5 medium containing Sorensen buffer and allowed to attach. Colloidal iron particles were prepared, as previously described [41], added at a final concentration of 1 mg mL^−1^ and carefully dispersed 30 min before infection. Cells were infected with *F. novicida* at MOI 20 and centrifuged at 100× *g* for 3 min at RT to synchronize infection. After 60 min of incubation at 27 °C, the infected cells were scraped off, transferred to a new tube, and washed twice with ice-cold Sorensen buffer and once with ice-cold HB buffer (0.5 mM Na2EGTA, 20 mM HEPES, 250 mM sucrose).

The pellet of infected cells was resuspended in ice-cold HB buffer supplemented with an EDTA-free protease inhibitor cocktail (Roche Diagnostic, Penzberg, Germany) and with 5 mg mL^−1^ Iodonitrotetrazolium Chloride (INT, Sigma-Aldrich, St. Louis, MO, USA). Cells were mechanically minced in a Dura-Grind stainless steel homogenizer (Dounce Dura-Grind^®^ Tissue Grinder; Braintree, Scientific, Inc., Waltham, MA, USA) and incubated with benzonase (50 units mL^−1^, Sigma-Aldrich, St. Louis, MO, USA) for 7 min at 37 °C. Nuclear and cellular debris were removed by centrifugation at 110× *g* for 5 min at 4 °C. The obtained postnuclear supernatant (PNS) was transferred to new tubes. HB buffer containing the protease inhibitor cocktail was added to the remaining pellet, mixed thoroughly, and centrifuged at 100× *g* for 3 min at 4 °C. PNS was passed through the MiniMACS column (OctoMACSTM Separation Unit; Miltenyi Biotec, Bergisch Gladbach, Germany) to remove the lysosomal and endosomal compartments loaded with colloidal iron. The flow-through fraction was carefully applied to 8 mL of 10% to 45% OptiPrep Gradient (Sigma-Aldrich, St. Louis, MO, USA) and centrifuged at 100,000× *g* at 4 °C for 2 h in the SW40 Swing Rotor (Beckman Coulter, Brea, CA, USA). The 800 µL fractions were collected from the top of the gradient and further analyzed. To determine the distribution of *F. novicida* in the gradient fractions, each fraction was plated on BCYE agar. After two days of incubation at 37 °C, the number of bacteria in each OptiPrep fraction was calculated.

### 2.3. SDS-PAGE and Western Blot

For Western blot analysis, cells were lysed in RIPA buffer (150-mM NaCl, 1% NP-40, 0.5% Na deoxycholate, 0.1% SDS, 50 mM Tris [pH 8.0]), with protease inhibitors (cOmplete, Roche, Basel, Switzerland), resuspended in 2× Laemmli buffer with β-mercaptoethanol (Carl Roth, Karlsruhe, Germany), and denatured for 6 min at 95 °C, and an equal protein amount was applied to 10% SDS-PAGE. The proteins were transferred to the nitrocellulose membrane (Bio-Rad Laboratories, Hercules, CA, USA). The membranes were blocked in 5% non-fat dry milk in 1× Tris-buffered saline—0.05% Tween 20 (TBS-T) for 1 h at room temperature. The following primary antibodies were used at subsequent concentrations: rabbit antibody against P80 (The Geneva Antibody Facility, Geneva, Switzerland)—1:1000, mouse monoclonal antibody against α-mitoporin (70-100-1)—1:2000, vacuolar ATPase subunit A (VatA, 221-35-2)—1:1000, protein disulfide isomerase (PDI, 221-64-1)—1:1000, a gift from M. Maniak. The membranes were incubated in primary antibodies overnight at +4 °C. Blots were washed for 30 min with TBS-T, and primary antibodies were detected using horseradish peroxidase-conjugated goat anti-mouse and goat anti-rabbit secondary antibodies diluted 1:1000 (Cell Signaling Technology, Inc., Danvers, MA, USA) and incubated at room temperature for 1 h, and again washed for 30 min. Enhanced chemiluminescence detection reagents Luminal Enhancer Solution (GE Healthcare, Buckinghamshire, UK) and Peroxide Solution (GE Healthcare, Buckinghamshire, UK) were used for the visualization of detected proteins by Chemi Doc XRR+ (Bio-Rad Laboratories, Hercules, CA, USA).

### 2.4. Transmission Electron Microscopy (TEM) 

Infected *D. discoideum* cells and isolated *Francisella novicida*-containing vacuoles were prepared for transmission electron microscopy according to the following protocol. *D. discoideum* cells were seeded in 24-well cell culture plates at a concentration of 106 cells mL^−1^ and allowed to adhere for 1 h. The cells were infected with *F. novicida* at MOI 20. At 15 min, 60 min, and 6 h after infection, the cells were washed with 1× Sorensen buffer, fixed with 2.5% glutaraldehyde (SPI Supplies, West Chester, PA, USA) for 30 min, and post-fixed with 1% osmium tetroxide for 30 min (SPI Supplies, West Chester, PA, USA) at 4 °C. The cells were dehydrated by ethanol series with increased concentrations, embedded in epoxy resin (SPI Supplies, West Chester, PA, USA), and polymerized for 24–48 h at 60 °C. Ultra-thin sections were cut. Infected cells, as well as the localization of intracellular bacteria, were observed by a Phillips Morgany transmission electron microscope (FEI, Waltham, MA, USA) using the following criteria: intact vacuolar membrane, damaged vacuolar membrane, and cytosol.

In addition, phagosome fractions were transferred to 24-well cell culture plates, washed with 1× Sorensen buffer, and prepared for TEM analysis, as described above.

### 2.5. Scanning Electron Microscopy (SEM)

After infection with *F. novicida*, the *D. discoideum* cells were examined with a scanning electron microscope (SEM). The cells were seeded in 24-well cell culture plates with coverslips coated with Alcian blue (Sigma-Aldrich, St. Louis, MO, USA) and allowed to adhere for 1 h. The cells were infected with *F. novicida* at MOI 20. After 15 and 60 min of infection, the cells were washed with Sorensen buffer and fixed with 1% glutaraldehyde and 0.5% paraformaldehyde for 1 h at 4 °C. Samples were post-fixed with 1% osmium tetroxide for 1 h at 4 °C and dehydrated with an ethanol series. The samples were dried with acetone and hexamethyldisilazane (SPI Supplies, West Chester, PA, USA). The samples were coated with carbon and analyzed using a JOEL scanning microscope (JOEL, Peabody, MA, USA).

### 2.6. Preparation of the Francisella novicida-Containing Fraction for LC-MS/MS Analysis

Four biological replicates of the *Francisella novicida*-containing fraction were prepared. The gradient fractions with the highest number of bacteria were collected, mixed with 13.5 M trichloroacetic acid (Sigma-Aldrich, St. Louis, MO, USA) at a ratio of 4:1 and incubated overnight at 4 °C. After protein precipitation overnight, protein pellets were centrifuged at 15,000× *g* (15 min, 4 °C) and washed three times with ice-cold 80% acetone in water. The protein pellets were dried and dissolved in 2% *w*/*v* sodium deoxycholate monohydrate (SDC, Sigma-Aldrich, St. Louis, MO, USA) containing 50 mM ammonium bicarbonate. Proteins were reduced with 1 mM dithiothreitol (Sigma-Aldrich, St. Louis, MO, USA) at 60 °C for 40 min and alkylated with 4 mM iodoacetamide (Sigma-Aldrich, St. Louis, MO, USA) at room temperature for 30 min. The excess of iodoacetamide was quenched by adding dithiothreitol, the concentration of SDC was adjusted to 1% with ammonium bicarbonate, and trypsin (Promega, Madison, WI, USA) was added for overnight digestion at 37 °C. The next day, the mixture was acidified and the precipitated deoxycholic acid was removed by extraction in ethyl acetate (Sigma-Aldrich, St. Louis, MO, USA). Samples were partially evaporated by vacuum centrifugation (Concentrator plus, Eppendorf, Hamburg, Germany) to remove residual ethyl acetate and then purified with C18 tips (100 µL, Pierce). Peptides were eluted with 65% methanol (Merck, Rahway, NJ, USA) in 0.1% trifluoroacetic acid (Thermo Fisher Scientific, Waltham, MA, USA). The elution fraction was partially evaporated and dissolved in 2% acetonitrile (Sigma-Aldrich, St. Louis, MO, USA) containing 0.1% trifluoroacetic acid and 3% acetic acid (Sigma-Aldrich, St. Louis, MO, USA). The samples were stored at −20 °C until analysis.

### 2.7. LC-MS/MS Data Acquisition

The LC-MS/MS system consisted of an Ultimate 3000 RSLC nano connected to a Q-Exactive mass spectrometer (both from Thermo Fisher Scientific, Waltham, MA, USA). A single injection of the peptide aliquot was analyzed for each biological replicate. Peptides were loaded onto a trap column and then separated with a gradient of 0.1% formic acid in 80% acetonitrile (solvent B) and 0.1% formic acid in water (solvent A) at a flow rate of 250 nl min−1 on a PepMap C18, 2 µm, 100 Å, 0.075 × 250 mm analytical column (Thermo Fisher Scientific, Waltham, MA, USA). The gradient profile was 0 to 5 min (4% B); 5 to 52 min (4–32% B); 52 to 75 min (32–55% B); 75 to 76 min (55–90% B); 76 to 86 min (90% B); 86 to 87 min (90–4% B); and 87 to 108 min (4% B). Full MS/Top7 data-dependent acquisition was used for peptide identification. Positive ion full-scan MS spectra from the 350 to 1650 m z^−1^ range were acquired with a 1 × 10^6^ target ion population in the Orbitrap at a resolution of 70,000 (at m z^−1^ 200). Precursor ions with assigned charge states 2–5, with a minimum threshold intensity of 5 × 10^4^ counts, and that had not fragmented in the previous 19 s were allowed to collisional dissociate at higher energy. Tandem mass spectra were recorded with a resolution of 17,500 and with other parameters such as 1 × 10^5^ for the target value of the automatic gain control, 100 ms for the maximum ion injection time, 1.6 m z^−1^ for the quadrupole isolation window, 27 for the normalized collision energy, and m z^−1^ 140 for the fixed first mass.

### 2.8. Proteomic Data Analysis

Peptides and proteins were identified by aligning raw files with the protein sequence database using the MaxQuant v1.6.17.0 computational platform [42]. The database contained protein sequences from *Dictyostelium discoideum* (12,735 entries, Uniprot), *F. tularensis* subsp. *novicida* strain U112 (1719 entries, Uniprot), and general contaminants (246 entries). The search parameters were as follows: Trypsin was used to digest proteins, two missed cleavages were allowed, carbamidomethylation (C) was set as a fixed modification, and oxidation (M), deamidation (N, Q), conversion of glutamine to pyroglutamic acid, and acetylation (protein N terminus) were selected as variable modifications. The option alignment between runs was enabled, and the other search settings were left at default. Data were visualized using Perseus v1.6.15.0 software [43]. Functional annotation enrichment analysis was performed using the tool DAVID 2021 (knowledgebase v2023q4) [44]. Enrichment of the gene ontology terms from the categories of biological process, molecular function, and cellular component was determined for FCV proteins against the background of a *Dictyostelium discoideum* proteome.

### 2.9. Transformation of D. discoideum

Plasmid DNA (pDXA-GFP Rab7a, a gift from *T. soldati*) was prepared using a commercially available plasmid preparation kit (Macherey-Nagel, City of Leicester, UK). The amount of DNA used per transformation was 10 µg. Transformation was performed using calcium phosphate precipitation, as described previously [45]. Briefly, *D. discoideum* cells were grown in cell plates in HL5 medium at 25 °C to a density of 1–2 × 10^6^ cells mL^−1^. The HL5 medium was replaced with Bis-Tris HL5, and the cells were incubated for 30 min. Bris-Tris HL5 medium contained 2.1 g Bis-Tris (Sigma-Aldrich, St. Louis, MO, USA), 10 g proteose peptone (Thermo Fischer Scientific, Waltham, MA, USA), 5 g yeast extract (Thermo Fischer Scientific, Waltham, MA, USA), and 10 g glucose (Merck, Hamburg, Germany) per liter of distilled water. During incubation, 10 µg of DNA solution was prepared in sterile water and 2× HBS solution [45]. The prepared DNA/HBS solution was mixed with 1.25 M CaCl_2_ and incubated for 30 min at RT. The Bis-Tris HL5 medium was removed, and the DNA solution was slowly added to the center of the plate. The plate was covered and incubated for 30 min while the *D. discoideum* cells took up the DNA. Without removing the DNA solution, Bis-Tris HL5 was added. The cells were incubated for 6 h to allow the cells to take up the DNA. After incubation, the medium was removed and 18% glycerol in HBS was added for 5 min. The glycerol solution was replaced with HL5 medium. After overnight incubation at 25 °C, G418 solution (Sigma-Aldrich, St. Louis, MO, USA) was added at a concentration of 10 µg mL^−1^. Transformants were selected for 10 days.

### 2.10. Confocal Laser Scanning Microscopy 

*D. discoideum* cells and *D. discoideum* cells expressing GFP-Rab7a (10^5^ cells mL^−1^) were seeded on 12 mm glass coverslips in 24-well cell culture plates and allowed to adhere. The cells were infected with *F. novicida* at MOI 20. At 15 min, 60 min, and 6 h postinfection, the coverslips were washed three times with ice-cold Sorensen buffer supplemented with 50 µM CaCl_2_ (SorC buffer, Sigma-Aldrich, St. Louis, MO, USA). Fixation and permeabilization were performed with ice-cold methanol for 30 min at −20 °C, followed by washing with SorC buffer and blocking with 3% bovine serum albumin (BSA, Sigma-Aldrich, St. Louis, MO, USA) for 30 min at RT. The cells were treated with primary goat monoclonal antibodies against *Francisella* (1:4000) and mouse monoclonal antibodies against vacuolar VatA (221-35-2, a gift from M. Maniak, 1:1000). They were then washed and incubated with secondary anti-mouse and anti-goat Alexa Fluor 488 and 555 antibodies (1:4000, Invitrogen, Waltham, MA, USA). The coverslips were mounted with Mowiol 4-88 mounting medium (Sigma-Aldrich, St. Louis, MO, USA). The cells were examined by confocal microscopy using an Olympus FV 1000 confocal laser scanning microscope (Olympus, Tokyo, Japan). Quantification of the colocalization of *F. novicida* with GFP-Rab7 and VatA in *D. discoideum* was performed manually by counting z-stack images (8 µM depth with 0.2 µM slices) of infected cells. Over 100 cells were counted for each condition, and experiments were performed in triplicate.

## 3. Results

### 3.1. F. novicida Resides in Intact Vacuoles of D. discoideum Cells

Previous studies have shown that *F. novicida* escapes from the phagosome and replicates in the cytosol after the infection of mammalian cells [37,38]. Lampe et al. have shown that *F. noatunensis* subsp. *noatunensis* replicates in the cytosol of infected *D. discoideum* cells [17]. In contrast, our previous results showed that *F. novicida* replicates in membrane-bound vacuoles in *H. vermiformis* [15]. To further investigate the localization of *F. novicida* in *D. discoideum*, as well as to define the time point for the isolation of *Francisella novicida*-containing vacuole, we used transmission and scanning electron microscopy.

**Figure 1:**
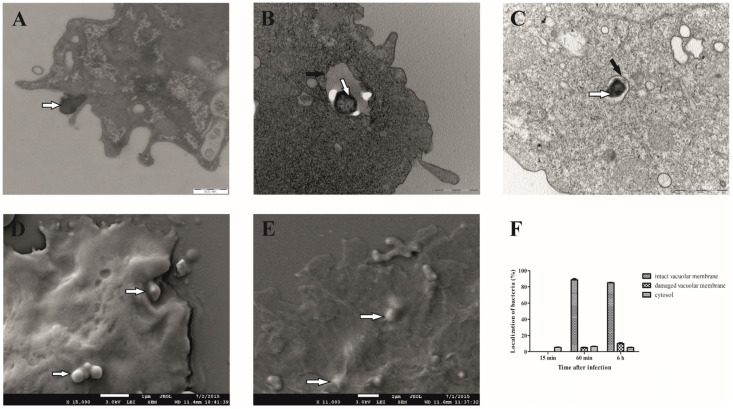
Electron microscopy of *D. discoideum* infected with *F. novicida*. The cells were infected with *F. novicida* at MOI 20 for 15 min (**A**), 60 min (**B**), and 6 h (**C**) and observed by TEM. Further, the cells were infected with *F. novicida* for 15 min (**D**) and 60 min (**E**) and observed using SEM. Quantitative results of localization of *F. novicida* within *D. discoideum*. Results were obtained by counting at least 100 bacteria for each sample (**F**). Black arrows show vacuolar membrane, and white arrows show bacteria. One representative micrograph out of three independent preparations is shown.

We observed that the bacteria begin to attach to the cell surface of *D. discoideum* 15 min after infection, which is manifested by the fact that *F. novicida* is partially surrounded by membrane-bound projections that are involved in the process of bacterial uptake (Figure 1A). After 60 min, the bacterium is localized in the cell within the membrane-bound vacuole (Figure 1B). Less than 10% of the bacterium is free in the cytosol or in a vacuole with a damaged membrane. After 6 h of infection, 85% of *F. novicida* are still localized in a vacuole with an intact membrane (Figure 1C). About 10% of the bacteria were localized in a damaged vacuole, and less than 5% of *F. novicida* were localized in the cytosol of *D. discoideum* (Figure 1F). These results clearly show that *F. novicida* reside in intact vacuoles after the infection of *D. discoideum* cells. Images obtained using SEM revealed that 15 min after infection, bacteria were localized on the surface of the infected cells (Figure 1D). However, after 60 min, bacteria were in the cell, confirming the intracellular localization of *F. novicida* (Figure 1E).

### 3.2. Isolation of Francisella novicida-Containing Vacuole (FCV) from Infected D. discoideum Cells

**Figure 2:**
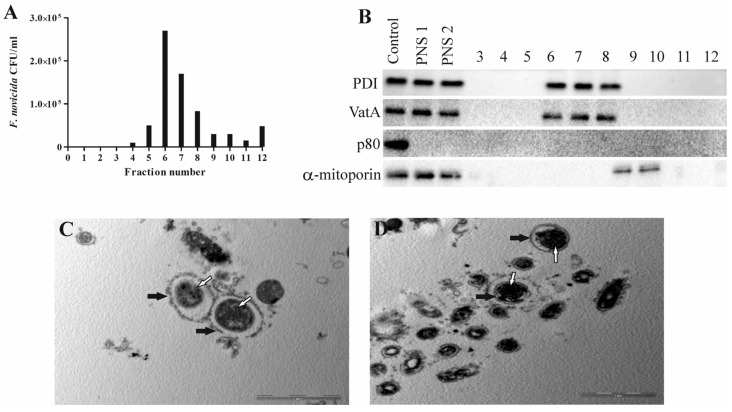
Successful isolation of *Francisella novicida*-containing vacuole from *D. discoideum*. An aliquot of each fraction was plated on BCYE agar, and CFU/mL of *F. novicida* in each 10–45% OptiPrep fraction was counted (**A**). For Western blot analyses, fractions 3 to 12, post-nuclear supernatant before (PNS1) and after (PNS2) magnetic separation were tested using the marker for α-mitoporin, PDI, p80, and VatA. *D. discoideum* lysate was used as a control (**B**). For TEM analyses of isolated *Francisella novicida*-containing vacuole, samples were washed and fixed with glutaraldehyde and osmium tetroxide. Ultra-thin sections were cut and observed using TEM. White arrows show vacuolar membrane, and black arrows show bacteria. One representative micrograph out of three independent preparations (**C**,**D**).

To evaluate the character of *Francisella novicida*-containing vacuoles, we developed, for the first time, a method to isolate FCV from *D. discoideum*. Based on results obtained using electron microscopy, isolation of the *Francisella novicida*-containing vacuole was performed 60 min after infection because most bacteria were localized within the membrane-bound vacuole at this time. The distribution of *F. novicida* in the gradients was determined by plating the OptiPrep fractions on BCYE agar plates and counting the bacterial CFU/mL. The results obtained show the enrichment of *F. novicida* in fraction 6 (Figure 2A). The fractions with the highest number of bacteria were processed for further analysis.

To determine whether the separation of the bacteria-containing compartments from the mitochondria was successful, we used Western blot and transmission electron microscopy. The distribution of the mitochondrial protein α-mitoporin in the OptiPrep fractions was used to localize the mitochondria and to indicate their separation from FCV. α-mitoporin was enriched in fractions 9 and 10 of the OptiPrep gradient, indicating the presence of mitochondria in these fractions (Figure 2B). In the isolation process, we included heavy labeling of mitochondria because of their density, which is similar to the density of vacuoles containing bacteria. However, the OptiPrep fractions were separated manually using pipet tips cut with a sterile blade to widen them and preserve FCV integrity. For that reason, contaminants from mitochondria are possible. Even though the α-mitoporin was not detected by Western blot in the fraction with the highest number of bacteria, several mitochondrial proteins were detected using mass spectrometry.

In postnuclear supernatant and fractions with the highest number of bacteria, the presence of PDI, an endoplasmic reticulum enzyme, was detected, suggesting the connection of FCV with these intracellular compartments (Figure 2B). Also, VatA was detected in fractions containing FCVs, suggesting acidification (Figure 2B). Protein p80, a late endosome membrane protein, was not detected in the postnuclear supernatant or isolated fractions 60 min after infection, demonstrating that it is not present in the endocytic compartment at this time point (Figure 2B).

Isolated FCV fractions were also prepared and analyzed by transmission electron microscopy. A low-magnification TEM image of the FCV-enriched fraction shows the purity of the fraction and freedom from other organelles (Figure 2C). The high-magnification TEM image of the FCV-enriched fraction shows a single bacterium localized in the membrane-bound vacuole (Figure 2D). TEM images also showed a damaged FCV membrane that we assume was damaged during the complex sample preparation steps.

### 3.3. The Proteome of Francisella novicida-Containing Vacuole Isolated from D. discoideum

To gain insight into the molecular processes during the first phase of *F. novicida* infection, the proteome composition of isolated FCV was characterized by LC-MS/MS. Four biological replicates were prepared, and the fractions with the highest number of bacteria were selected for proteomic analysis. After filtering out proteins representing contaminants (e.g., keratins, serum proteins), we identified 1025 proteins present in all 4 replicates (Table S1). Of these, 689 proteins contained label-free quantification intensities and were considered for further analysis. Most of these proteins were host proteins (684 proteins, Table S2), and 5 of them were identified as *F. novicida* proteins (Table S3). To identify biological themes in the FCV proteome, functional annotation enrichment analysis of gene ontology terms from the categories of biological process, molecular function, and cellular component was performed (Table S4). The selected enriched terms for each category are shown in Table 1. Accordingly, the host proteins found in the FCV proteome were clustered into the following terms, e.g., phagocytic vesicle, extracellular matrix, mitochondrion, and ribosome for the cellular component, e.g., translation, response to bacterium and phagocytosis for the biological process, and e.g., structural constituent of ribosome, oxidoreductase activity, structural constituent of cytoskeleton and protein binding for the molecular function. The individual proteins assigned to terms phagocytic vesicle, response to bacterium, phagocytosis, and protein binding are listed in Table S4. Term phagocytic vesicle, represented by 189 proteins, confirms FCV isolation and enrichment from *D. discoideum*. Similarly, terms mitochondrion, ribosome, and proteasome core complex (Table S4) indicate the presence of non-targeted components in the FCV isolates. In addition, the terms response to bacterium and phagocytosis indicate that the process of initial interaction of host cells with bacteria is captured within the data. Proteomic analyses of FCV isolated from *D. discoideum* 60 min after infection generally show that FCV communicates with proteins involved in different processes and compartments after infection.

### 3.4. Colocalization of Rab GTPase and Vacuolar H+-ATPase Subunit A with F. novicida in D. discoideum

After infection of *D. discoideum*, *F. novicida* is localized in the early vacuolar compartment, which is characterized by rapid acidification. Acidification is essential for the maturation of the compartments of the endocytic pathway, and one of the best characterized complexes delivered to the phagosome in the early stages of infection is vacuolar ATPase. In addition, small GTPases are an important factor in phagosome maturation [46]. Among the 684 host cell proteins identified in the FCV proteome of Dictyostelium, 13 small GTPases (DDB_G0268034, Rab11A, Rab11C, Rab14, Rab1B, Rab2B, Rab7A, Rab8A, RabC, Ranbp1, RapA, RasG, and Rac1A) and 4 vacuolar H+-ATPase subunits (VatM, VatB, VatA, and VatE) were identified (Table S4).

**Figure 3:**
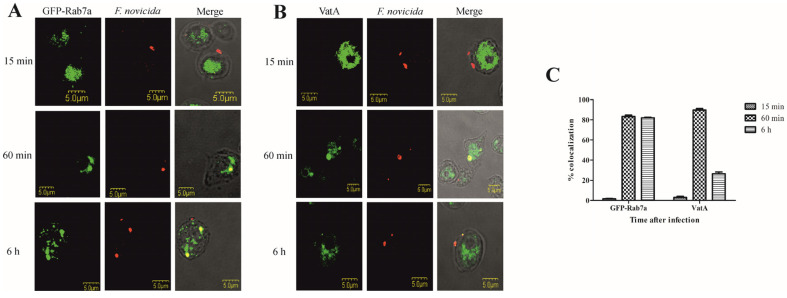
Colocalization of Rab7a and VatA with *F. novicida* in *D. discoideum*. Representative confocal laser scanning microscopy images of *D. discoideum* cells and *D. discoideum* cells expressing the GFP-Rab7a (**A**) and VatA (**B**) infected with *F. novicida* at MOI 20 for 15 min, 60 min, and 6 h. Quantitative analyses of colocalization of *F. novicida* with GFP-Rab7 and VatA in *D. discoideum*. The results shown are representative of three independent experiments and displayed as a mean with standard deviation (error bars) of triplicate samples (**C**).

To validate the results of the FCV proteome study, we focused on the characterization of the Rab GTPase Rab7a and the catalytic subunit A of the V-type proton ATPase (VatA) by confocal microscopy. Localization of Rab GTPases was examined by transformation of *D. discoideum* with GFP fusion proteins, and VatA was visualized with a monoclonal antibody against VatA. *D. discoideum* cells and transfected *D. discoideum* fusing GFP with Rab7a were infected with *F. novicida*. Representative images of amoebae expressing Rab7a-GFP show that *F. novicida* does not colocalize with GFP-Rab7a in *D. discoideum* 15 min after infection (Figure 3A). At 60 min postinfection, more than 80% of *F. novicida* colocalized with GFP-Rab7a (Figure 3A,C). Six hours after infection, *F. novicida* colocalized with GFP-Rab7a, confirming the localization of the bacteria in the vacuole (Figure 3A). We quantified the colocalization of Rab7a-GFP and *F. novicida* 6 h postinfection and found that ~80% of intracellular bacteria colocalize with GFP-Rab7a. Using confocal microscopy, we confirmed the proteomic findings that Rab7a accumulates on FCV after 60 min of infection.

Colocalization of *F. novicida* with vacuolar VatA was followed for 15 min, 60 min, and 6 h after infection of *D. discoideum*. Our results showed that 15 min after infection of *D. discoideum* cells with *F. novicida*, only 3% of bacteria colocalize with VatA (Figure 3B,C). After 60 min, 89% of *F. novicida* colocalize with VatA (Figure 3B,C). The results also show that 6 h after infection, 26% of *F. novicida* colocalize with VatA, indicating the presence of bacteria in non-acidified vacuoles (Figure 3B,C).

## 4. Discussion

The ability of *Francisella* to successfully establish infection and multiply in a variety of cells is reconciled with the ability of bacteria to establish and multiply in a vacuolar compartment in amoeboid cells or to escape from the original vacuolar compartment after infection and multiply in the cytosol of mammals. For pathogenic bacteria, intracellular compartments provide protection from cellular and humoral components of the mammalian immune system. In addition, the bacteria have access to various nutrient sources.

*D. discoideum* is an established model organism for studying the intracellular life cycle and virulence factors of many pathogenic bacteria. The amoeba has also been used as a model for isolating bacteria-containing compartments that form after infection. Lampe et al. showed that the infection of *D. discoideum* with *F. noatunensis* recapitulates the cellular aspects of *Francisella* infection of mammalian macrophages [17]. In contrast, *F. novicida* survives after invading *D. discoideum* and forms a replicable FCV, suggesting that *D. discoideum* could serve as a model organism to study the intracellular aspects of *F. novicida* infection [18]. Our results showed that the intracellular life cycle of *F. novicida* in *D. discoideum* is similar to the intracellular life cycle of *Francisella* spp. in other amoebae. In addition, we showed that *F. novicida* localized to the membrane-bound vacuole 6 h after infection, and approximately 15% of the bacteria localized to the damaged vacuole or cytosol of *D. discoideum*.

To study the sensitive intracellular compartments containing bacteria, we have developed a method to isolate FCV from *D. discoideum*. The isolation is based on the elimination of extracellular bacteria by several washing steps, lysis of infected cells, and fractionation on a density gradient matrix. Successful isolation of FCV and separation from other intracellular compartments was confirmed by Western blot and TEM.

To evaluate the character of FCV, we analyzed the host proteins involved in the formation of these compartments after 1 h of *D. discoideum* infection. We reproducibly identified a set of 684 proteins in 4 biological replicates. Using functional annotation enrichment analysis, we found the terms phagocytic vesicle, response to bacterium, and phagocytosis to be significantly enriched in the FCV proteome, directly linking the identified proteins to the biology of the host cell infection. On the other hand, the data indicated contamination of the FCV isolate with non-phagosomal proteins, particularly ribosomal and mitochondrial proteins. We hypothesize that some of these proteins may also be present as a result of the communication of *Francisella novicida*-containing vacuoles with other compartments after infection. Of the known vacuolar proteins, we detected several small GTPases and vacuolar H+-ATPase subunits involved in phagosome migration and maturation. As expected, we detected *Francisella* proteins in the FCV proteome. However, we detected very few *Francisella* proteins, which were characterized as low abundance and, therefore, hidden in the *D. discoideum* proteome.

A proteomic study of *F. tularensis* LVS-containing phagosomes was reported from infected Bcg/Nramp1 congenic B10R and B10S macrophages [47]. Protein-2-DE patterns of *F. tularensis*-containing compartments identified *F. tularensis* 60 kDa chaperonin and hypothetical 23 kDa protein based on the criterion that they are present in B10R-FCP, but absent in B10S-FCP gels [47]. A major challenge in studying host–pathogen interactions is the limited material, especially because bacterial proteins outnumber identified host proteins that are unlikely to be involved in the infection process [48]. In this study, we identified five *Francisella* proteins in the isolated FCV vacuole 60 min after infection. As in a previous study, we also identified 60 kDa of a chaperonin in the proteome of FCV isolated from *D. discoideum. F. tularensis* GroEL has been shown to localize to the bacterial surface, and its expression is increased in response to stress stimuli such as heat and hydrogen peroxide [49,50].

Other pathogenic bacteria such as *Mycobacterium marinum* and *L. pneumophila* are also located in a membrane-bound compartment in *D. discoideum* [51]. To avoid acidosis, vacuolar ATPases of *Legionella*- and *Mycobacteria*-containing vacuoles are excluded [52]. Previous studies have shown that *F. noatunensis*-containing phagosomes in *D. discoideum* are associated with the V-ATPase subunit VatA, suggesting acidification [17]. We detected VatA by mass spectrometry and confirmed its presence by Western blot and confocal microscopy.

The small GTPases are involved in vesicle transport and localized to the membrane of the endosomal-lysosomal compartment. The host factors identified in the FCV proteome included a total of 13 small GTPases, of which Rab7a was validated by confocal microscopy. In mammalian cells, the fusion of late endosomes with lysosomes is regulated by the GTPase Rab7 [53]. As *F. tularensis* escapes into the cytosol after the infection of mammalian cells, Rab7 is transiently recruited to FCV at 15 and 30 min postinfection and is lost at 60 min postinfection [53]. In *D. discoideum*, Rab7 rapidly associates with early phagosomes and is responsible for transporting lysosomal enzymes and membrane proteins to early phagosomes [54]. The colocalization of *F. novicida* with GFP-Rab7a suggests that it plays an important role in the interaction of early phagosomes with other compartments of the endosomal-lysosomal system. Similar to our results, the Ras-related protein Rab7a was also detected in vacuoles containing *Legionella*, *Salmonella*, *Chlamydia*, *Simcania*, and *Mycobacteria* isolated from HeLa and THP1 cells, RAW264.7 macrophages, and *D. discoideum* (reviewed in [48]). Consistent with the identification of Rab GTPases in the vacuole of the pathogen in our study, previous studies have also identified Rab proteins and shown that Arf1, Rab8a, Rab 10, and Rab32 promote the intracellular growth of *L. pneumophila*, whereas Rab5a, Rab14, and Rab21 restrict intracellular replication [30].

Studies have shown that *L. pneumophila*, after entry into the host cells, creates a unique endoplasmic reticulum (ER)-like organelle that supports intracellular replication [55,56,57,58]. In the proteome of FCV isolated from *D. discoideum* after 60 min of infection, we identified several ER proteins, and Western blot analyses showed that PDI is localized in the fractions containing FCVs, suggesting the interaction of these compartments. However, further studies of interactions between vacuoles containing *F. novicida* and the host ER should be conducted.

Overall, in this study, we showed that *F. novicida* survives in *D. discoideum* in the membrane-bound vacuole. To better understand FCV formation and interaction with other cellular compartments, we developed a method to isolate pathogen-containing vacuoles and characterize them by mass spectrometry. Proteomic analyses of FCV isolated from amoebae 60 min postinfection indicate that pathogenic vacuole formation requires interaction with multiple cellular compartments and metabolic pathways. The identification of 13 Rab family small GTPases and 4 vacuolar ATPase subunits and the characterization of Rab7a and VatA by confocal microscopy indicate their role in host–pathogen interaction after infection. The results of this study shed light on the mechanisms *Francisella* employs for intracellular survival in phagocytic amoebae.

## Figures and Tables

**Table 1:** List of GO terms. Selected gene ontology (GO) terms enriched in the FCV proteome of *D. discoideum*. Cellular component (CC), Biological process (BP), Molecular function (MF). * Proteins assigned to these terms are listed separately in Table S4.

GO Category	Term Description	Gene Count	Fold Enrichment	False Discovery Rate
CC	phagocytic vesicle *	189	6.5	1.7 × 10^−115^
CC	extracellular matrix	92	6.3	6.5 × 10^−51^
CC	mitochondrion	119	3.2	5.0 × 10^−31^
CC	ribosome	59	6.1	1.2 × 10^−30^
BP	translation	69	4.4	2.8 × 10^−25^
BP	response to bacterium *	46	4.9	2.1 × 10^−18^
BP	tricarboxylic acid cycle	22	8.0	1.8 × 10^−13^
BP	phagocytosis *	34	3.4	1.4 × 10^−08^
MF	structural constituent of ribosome	58	4.9	8.1 × 10^−24^
MF	oxidoreductase activity	91	2.5	4.3 × 10^−15^
MF	structural constituent of cytoskeleton	21	6.1	6.7 × 10
MF	protein binding *	66	2.0	2.3 × 10^−06^

## Data Availability

The original contributions presented in the study are included in the article/Supplementary Material; further inquiries can be directed to the corresponding author.

## Supplementary Materials

The following supporting information can be downloaded at https://www.mdpi.com/article/10.3390/microorganisms12101949/s1, Table S1: Complete list of identified proteins on FCV isolated from *D. discoideum* 60 min after infection; Table S2: List of *D. discoideum* proteins considered for FCV functional annotation enrichment analysis; Table S3: *F. novicida* proteins identified on FCV isolated from *D. discoideum* cells 60 min after infection; Table S4: Results of functional annotation enrichment analysis of *D. discoideum* FCV proteins.
